# Berberine attenuates septic cardiomyopathy by inhibiting TLR4/NF-κB signalling in rats

**DOI:** 10.1080/13880209.2021.1877736

**Published:** 2021-02-04

**Authors:** Huiqi Chen, Qian Liu, Xiangqi Liu, Jinlan Jin

**Affiliations:** a Department of Ultrasonography, Guangzhou Red Cross Hospital, Medical College, Jinan University, Guangzhou, China; b Department of Cardiology, The Second Affiliated Hospital, University of South, Hengyang, China; c Department of Critical Care Medicine, Shenzhen Hospital (Futian) of Guangzhou University of Chinese Medicine, Shenzhen, China

**Keywords:** Sepsis, hemodynamics, p65, myocardial dysfunction, traditional Chinese medicine

## Abstract

**Context:**

Berberine (Ber) can increase the survival rate of septic mice and inhibit inflammation, but whether it has a protective effect on septic cardiomyopathy (SCM) is unclear.

**Objective:**

To investigate whether Ber ameliorates SCM in a rat model and its potential mechanism.

**Materials and methods:**

Male SD rats were randomly divided into three groups: control (Con, *n* = 6) (DD H_2_O, 2 mL/100 g, ig, qd × 3 d, then saline, 10 mg/kg, ip); sepsis [LPS (lipopolysaccharide), *n* = 18] (LPS 10 mg/kg instead of saline, ip); and berberine intervention (Ber, *n* = 18) (Ber, 50 mg/kg instead of DD H_2_O, ig, qd × 3 d, LPS instead of saline, ip). Hemodynamics, HE staining, ELISA and western blot were performed at 6, 24, and 48 h after intraperitoneal injection of LPS to evaluate the effect of berberine in septic rats.

**Result:**

Berberine could recover myocardial injury by partially increased ± dp/dt max (1151, 445 mmHg/s) and LVEDP levels (1.49 mmHg) with LPS-induced rats, as well as an ameliorated increase of cTnT (217.53 pg/mL) in the Ber group compared with that in the LPS group (at 24 h). In addition, HE staining results showed that berberine attenuated the myocardial cell swelling induced by LPS. In contrast to the LPS group, the up-regulation of TLR4, p65 TNF-α, and IL-1β were attenuated in the Ber group.

**Discussion and conclusions:**

Berberine showed a protective effect on septic cardiomyopathy rats possibly through inhibiting the activation of TLR4/NF-κB signalling pathway. Whether it improves SCM through other mechanisms is our ongoing research.

## Introduction

Sepsis is a serious complication in acutely ill patients with infection or trauma, and could lead to life-threatening organ dysfunction from dysregulated host response to infection. Sepsis-induced multiple organ failure, especially septic cardiomyopathy (SCM), occurs in 40–60% of patients with septic shock (Maeder et al. 2006). Conventional anti-infective and support therapies failed to reduce the mortality in sepsis patients, and mortality rates in patients with SCM are high (Freifeld et al. 2011; Ullmann et al. 2012). Effective SCM prevention is the key to reducing mortality rates in sepsis patients (Cirulis et al. 2019). The mechanism of SCM remains unclear, and effective targeted treatments for SCM are still lacking.

SCM, with three main characteristics of ventricular dilatation, decreased left ventricular ejection fraction and cardiac dysfunction recovery within 7–19 days, is induced by endotoxins and cytokines (Sato and Nasu 2015). It was reported that ventricular dilatation and decreased left ventricular ejection fraction are the earliest parameters for SCM evaluation. Sepsis-induced myocardial damage is closely related to reduced myocardial energy supply and weakened myocardial contractility (Kimchi et al. 1984; Chu et al. 2016). Abnormal biomarkers, including troponin (cTnI/cTnT) and natriuretic peptides (NPs), are potential indicators of cardiac dysfunction. The level of cTnI/cTnT usually increases with the severity of the SCM disease (Kimchi et al. 1984; Maeder et al. 2006; Freifeld et al. 2011; Ullmann et al. 2012; Sato and Nasu 2015; Cheng and Regnier 2016; Chu et al. 2016; Cirulis et al. 2019). A large number of inflammatory factors, including tumour necrosis factor alpha (TNF-α), interleukin 6 (IL-6), IL-8, and IL-1β, are also involved in sepsis-induced myocardial injury (Hobai et al. 2015).

The toll-like receptors 4 (TLR-4)/NF-κB signalling pathway plays a crucial role in the regulation of myocardial damage caused by sepsis. TLR-4 mediates Myd88-dependent activation of the NF-κB signalling pathway and releases downstream inflammatory factors, including TNF-α, IL-6, IL-8 and IL-1β, which play a role in the subsequent sepsis-related inflammatory response (Szostak et al. 2015). When sepsis occurs, LPS in the cell wall of Gram-negative bacteria activates the intrinsic immune recognition of TLR-4, which in turn activates IL-1 receptor-associated kinase (IRAK1) and tumour-necrosis factor (TNF) receptor-associated factor 6 (TRAF6) to transduce extracellular signals. The transcription of target genes and release of downstream inflammatory factors, including TNF-α and IL-6, are subsequently initiated by NF-κB (Chu et al. 2016). TNF-α and IL-6 further affect the mitochondrial function and activate intracellular signal transduction to inhibit the transport of myocardium Ca^2+^, resulting in myocardial dysfunction (Jiang et al. 2014).

Berberine (C_20_H_19_NO_5_) is an isoquinoline alkaloid isolated from the traditional Chinese medicines *Coptis chinensis* Franch (Ranunculaceae) and *Phellodendron* (Rutaceae), which have been used to treat gastroenteritis for 1400 years. Recent studies have found that berberine increases the survival rate of sepsis mice by affecting the extracellular release of high mobility group box 1 (HMGB1) (Lee et al. 2013). Berberine was also found to inhibit LPS-induced cardiac dysfunction (Denk et al. 2012). Furthermore, berberine reduces the release of the cytokines TNF-α and IL-1β by inhibiting IκB in lung tissue cells (Lee et al. 2007). Berberine is also considered a potential anticancer drug for the treatment of endometrial cancer, as it suppresses growth and metastasis via miR-101 (Wang and Zhang 2018). Berberine also inhibits the activation and expression of transcription factor in sepsis by downregulating the NF-κB, Akt and MAPK/JNK/p38/ERK pathways (Gao et al. 2014).

However, the role of berberine in protecting SCM remains unknown. In this study, a lipopolysaccharide (LPS)-induced SCM rat model was established to assess the effect of berberine on SCM and investigate its impact on the TLR/NF-κB signalling pathway.

## Materials and methods

### Animal experiments

Male Sprague Dawley rats, weighing 250–300 g, were provided by Guangdong Medical Lab Animal Centre (Animal Qualification Number: SCXK 2013-0002) and housed under a 12 h light/dark cycle environment, with the laboratory environment temperature controlled at 25 ± 1 °C, relative humidity 55–65%, 5 rats/cage, with free access to eat and drink. The animals were allowed to adapt to the surrounding environment 1 week before the study. The animal experiments were approved by the Ethics Committee of Guangzhou Red Cross Hospital.

The rats were randomly divided into three groups, including the control (Con, *n* = 6), sepsis (LPS, *n* = 18), and berberine intervention (Ber, *n* = 18) groups. The LPS group and the Ber group were administered intragastrically with double-distilled water (2 mL/100 g) and berberine (50 mg/kg, Sigma-Aldrich, USA) (Li et al. 2012), respectively, once a day for 3 days before LPS (10 mg/kg, Sigma-Aldrich, USA) (de Pádua Lúcio et al. 2018) was injected intraperitoneally upon the 3rd day after intragastric administration for 1 h (Wang et al. 2019). In the control group, double-distilled water (2 mL/100 g) was administered intragastrically for 3 days before saline (1 mL/100 g) was injected instead of LPS.

The LPS group and the Ber groups were further divided into 3 subgroups (*n* = 6), respectively, for follow-up experiments at 6, 24, and 48 h after intraperitoneal injection of LPS. After the hemodynamic experiments were finished, the blood was collected, and then rats were sacrificed by cervical dislocation at these time points, the myocardial tissues samples were collected for later use. Additional gavage of berberine or double-distilled water was applied once 24 h after rats were injected intraperitoneally to relevant 48 h subgroups. Since detection indexes in the control group showed no significant differences over time, we used 6 h in this group as baseline for the LPS and Ber groups.

### Cardiac hemodynamics

At 6, 24, and 48 h, respectively, rats were intraperitoneally injected with 3% pentobarbital sodium (Sinopec Group Chemical Reagent Co., Ltd., China) for anaesthesia and intubated for ventilation assistance. The BL-420F biological signal acquisition and analysis system (Chengdu Taimeng Technology Co., Ltd., China) was prepared by connection to a PT-100 biological blood pressure sensor (Chengdu Taimeng Technology Co., Ltd., China) and a PE50 polyethylene tube. The right common carotid artery was isolated, and the distal end of the common carotid artery was ligated. A polyethylene tube was slowly inserted into the common carotid artery towards the heart to observe the pressure waveform on the monitor. After stabilising the waveform, left ventricular systolic pressure (LVSP), left ventricular end-diastolic pressure (LVEDP), and maximum rate of left ventricular pressure development and decay (± dp/dt max) were recorded.

### Enzyme-linked immunosorbent assay (ELISA) assay for detecting markers of myocardial injury and inflammatory response

Totally 2 mL of blood was obtained and centrifuged at 4 °C for 10 min at 3,000 rpm to collect the supernatant which was immediately stored at −20 °C. An ELISA kit for rat plasma cardiac troponin T (cTnT) (Wuhan Huamei Biotechnology Co., Ltd., China) was used for detecting the levels of cTnT, according to the manufacturer’s instructions. Next, rats were killed by cervical dislocation, and tissues from the left ventricular myocardial part of the heart were collected and homogenized with the highly efficient radio immunoprecipitation assay (RIPA) tissue/cell lysis buffer (Beijing Solarbio Science & Technology Co., Ltd., China). ELISA kits for rat myocardial tissue TNF-α and IL-1β (Wuhan Huamei Biotechnology Co., Ltd., China) were adopted to detect the amounts of TNF-α and IL-1β, respectively, according to the manufacturer’s instructions. Optical density in each well was measured on a microplate reader (Promega, USA) at a wavelength of 450 nm, and the corresponding concentration was calculated.

### DNA-binding ELISA assays to measure NF-κB activation

Myocardial nuclear proteins were extracted with Nuclear Extract Kit (Active Motif, Inc., USA). Then, NF-κB p65 Transcription Factor Assay Kits (TransAM; Active motif, Inc., USA) was used to detect nuclear p65 content, according to the manufacturer’s instructions. Sample (20 μL of the specimen diluted in complete lysis buffer), positive control (5 μL of the provided Raji nuclear extract diluted in 15 μL complete lysis buffer), and blank (20 μL complete lysis buffer) wells were incubated for 1 h at room temperature with mild agitation at 100 rpm. After washing for three times with 200 μL of 1X wash buffer, 100 μL of diluted anti-NF-κB p65 antibodies (1:1000) was added and incubated for 1 h. Thereafter, 100 μL of horseradish peroxidase (HRP)-conjugated secondary antibody (1:1000) was added and incubated for 1 h. After washing for four times, each well was supplemented with 100 μL developing solution and incubated for 2 min away from light. Finally, 100 μL of stop solution was added, and absorbance was read within 5 min at a wavelength of 450 nm.

### Haematoxylin and eosin staining

The apical part of the myocardial tissue was collected and fixed with 4% neutral formaldehyde. The tissues samples were then embedded in paraffin and sectioned at 4 μm slices. The slides incubated with haematoxylin underwent nuclear staining, and were treated with 0.5% hydrochloric acid alcohol and stained with 1% eosin at room temperate for about 3 min. The haematoxylin and eosin (HE)-stained sections were observed under light microscopy at 20× magnification.

### Western blot

Myocardial tissue homogenates were obtained as described above. The lysates were centrifuged at 5000 *g* at 4 °C for 5 min and the resulting supernatants were stored at −80 °C until use. Proteins were extracted strictly according to instructions accompanying the cytoplasmic protein kit (Solarbio, China). TLR4 protein expression in the myocardial tissue was detected by Western blot. Total protein was separated by Sodium Dodecyl Sulfate-Polyacrylamide Gel Electrophoresis (SDS-PAGE) and transferred onto a polyvinylidene difluoride (PVDF) membranes (Millipore, Billerica, MA, USA). The membranes were blocked with 5% skim milk, followed by successive incubations with rabbit anti-mouse TLR4 polyclonal antibody (1:2000, Santa Cruz, USA) at 4 °C overnight and mouse anti-rabbit IgG monoclonal secondary antibody (1:10000, Bioss, China) for 2 h. The membranes were next incubated with ECL ultra western HRP substrate (Millipore, Billerica, MA, USA) and detected on a gel imaging system (ImageQuant LAS 4000, GE Healthcare, USA). Glyceraldehyde 3-phosphate dehydrogenase (GAPDH) (CST, USA, diluted, 1:1000) was used as a reference control.

### Statistical analysis

All the data were expressed as mean ± standard deviation. One-way analysis of variance (ANOVA) was used for comparison among the three groups, and pairwise comparison was performed by Student-Newman-Keuls (SNK) analysis. The associations of plasma cTnT levels with TLR4, p65, TNF-α, and IL-1β amounts were evaluated by Pearson correlation analysis. Statistical analyses were performed with SPSS v20.0 (Chicago, IL, USA), and *p* < 0.05 was considered statistically significant.

## Results

### Berberine improves cardiac hemodynamics in a rat sepsis model

**Figure 1.**
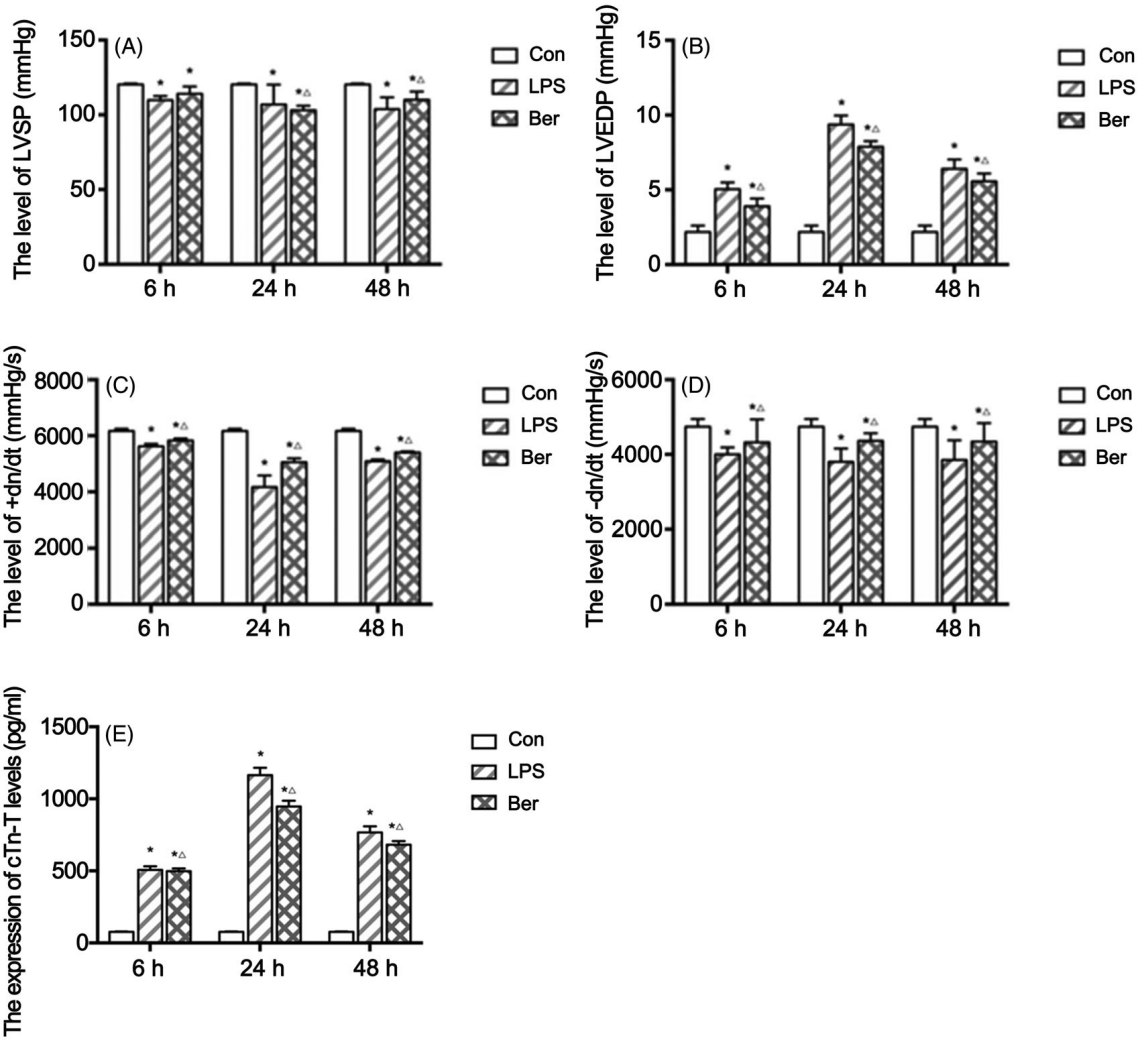
Effect of berberine on cardiac hemodynamics in a rat model of sepsis. (A) LVSP (mmHg) at 6, 24, and 48 h; (B) LVEDP (mmHg) at 6, 24, and 48 h; (C) +dp/dt max (mmHg) at 6, 24, and 48 h; (D) –dp/dt max (mmHg) at 6, 24, and 48 h. (E) Levels of cTnT measured by ELISA at 6, 24, and 48 h. *p < 0.05 indicates significance in comparison with the Con group; △*p* < 0.05 indicates significance in comparison with the LPS group.

The LPS group showed lower level LVSP (109.68 ± 2.71 mmHg, 106.87 ± 13.14 mmHg, 103.68 ± 7.85 mmHg at 6, 24, and 48 h, correspondingly) (Figure 1(A)) and ± dp/dt max (+dp/dt: 5623.37 ± 87.82 mmHg/s, 4172.78 ± 408.17 mmHg/s, 5088.30 ± 66.27 mmHg/s at 6, 24, and 48 h, correspondingly; –dp/dt: 4002.88 ± 185.22 mmHg/s, 801.48 ± 356.62 mmHg/s, 3853.35 ± 522.96 mmHg/s at 6, 24, and 48 h, correspondingly) (Figure 1(C, D)), and higher LVEDP (5.05 ± 0.44 mmHg, 9.37 ± 0.60 mmHg, 6.40 ± 0.63 mmHg at 6, 24, and 48 h, correspondingly) (Figure 1(B)) compared with the control group (LVSP: 118.99 ± 3.07 mmHg at 6 h; +dp/dt: 6131.76 ± 100.44 mmHg at 6 h; –dp/dt: 4818.70 ± 241.52 mmHg/s at 6 h; LVEDP: 2.18 ± 0.43 mmHg at 6 h) (all *p* < 0.05). In the Ber group, ± dp/dt max was significantly higher (+dp/dt: 5831.92 ± 71.26 mmHg/s, 5057.78 ± 139.53 mmHg/s, 5405.68 ± 38.36 mmHg/s at 6, 24, and 48 h, correspondingly; –dp/dt: 4323.47 ± 615.70 mmHg/s, 4363.02 ± 203.79 mmHg/s, 4341.65 ± 497.10 mmHg/s at 6, 24, and 48 h, correspondingly) than that of the LPS group at 6, 24, and 48 h (all *p* < 0.05) (Figure 1(C, D)), while LVSP was only higher at 24 h (103.03 ± 2.97 mmHg) and 48 h (110.05 ± 5.53 mmHg) (all *p* < 0.05) (Figure 1(A)). LVEDP was significantly lower in the Ber group (3.90 ± 0.52 mmHg, 7.88 ± 0.37 mmHg, 5.57 ± 0.52 mmHg at 6, 24, and 48 h) compared with the LPS group at 6, 24, and 48 h (all *p* < 0.05) (Figure 1(B)). These results suggested that berberine inhibited diastolic function reduction and improved cardiac function, and might also stimulate myocardial contraction, in rats administered LPS.

To investigate myocardial injury in rats with sepsis, cTnT levels were measured by ELISA. As shown in Figure 1(E), cTnT levels in the LPS group (506.60 ± 25.66 pg/mL, 1164.71 ± 51.29 pg/mL, 767.29 ± 42.04 pg/mL at 6, 24, and 48 h, correspondingly) were increased at each time point compared with that in Con group (76.61 ± 3.29 pg/mL at 6 h) (all *p* < 0.05) and peaked at 24 h. This, combined with the hemodynamics data described above, indicated the rat SCM model was successfully established. Meanwhile, cTnT levels in the Ber group were significantly reduced (498.36 ± 18.32 pg/mL, 947.18 ± 40.28 pg/mL, 682.13 ± 25.05 pg/mL at 6, 24, and 48 h, correspondingly) compared with those of the LPS group at 6, 24, and 48 h (all *p* < 0.05). This finding suggested that berberine reduced myocardial injury in SCM induced by LPS in rats.

### Berberine reduces cardiomyocyte injury and inflammatory cell infiltration

**Figure 2.**
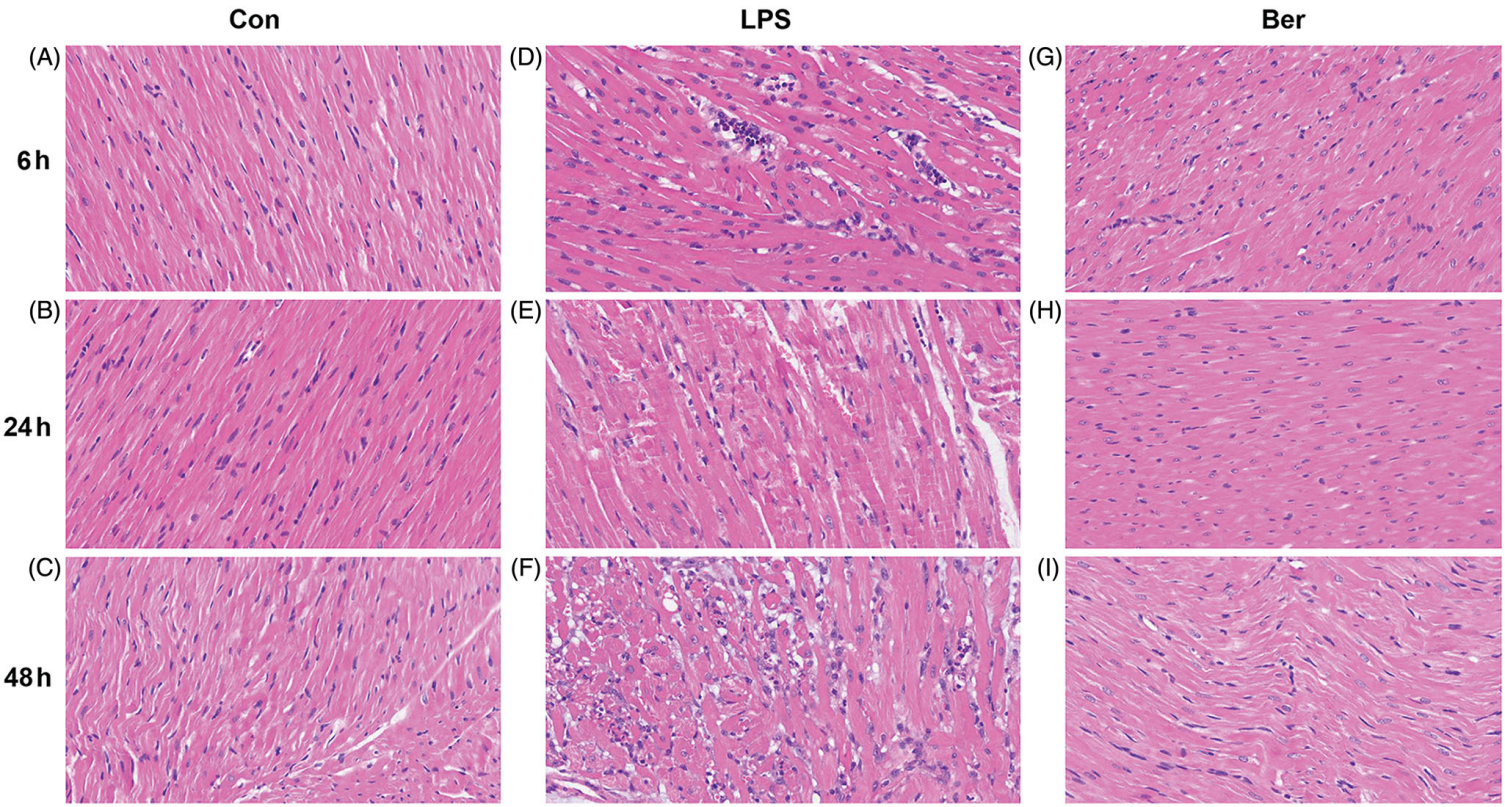
Histological changes of the myocardial tissue assessed by H&E staining. (A, B, C) Histopathological features of the myocardial tissue in the Con group at 6, 24, and 48 h. (D, E, F) Cardiomyocytes were swollen and enlarged, with infiltrated lymphocytes emerging in the LPS group at 6, 24, and 48 h. (G, H, I) Histopathological features of the myocardial tissue in the Ber group at 6, 24, and 48 h. Magnification, 200X.

To assess the effect of berberine on myocardial injury in rats with LPS-induced sepsis, we performed pathological H&E staining. The results showed that cardiomyocytes were swollen and enlarged in the LPS group compared with control rats. Infiltrated lymphocytes emerged as early as at the 6 h time point, and severely increased at 24 and 48 h (Figure 2). Compared with the LPS group, the Ber group had smaller cardiomyocytes and decreased amounts of infiltrated lymphocytes across the three time points (Figure 2). These findings suggested that berberine alleviated myocardial cell swelling induced by the infiltration of lymphocytes or neutrophils, as well as myocardial fibre fracture, especially at 24 h, and extenuated LPS-induced cardiomyocyte injury and inflammatory cell infiltration in SCM rats.

### Berberine reduces TLR4 protein expression in the myocardial tissue

**Figure 3.**
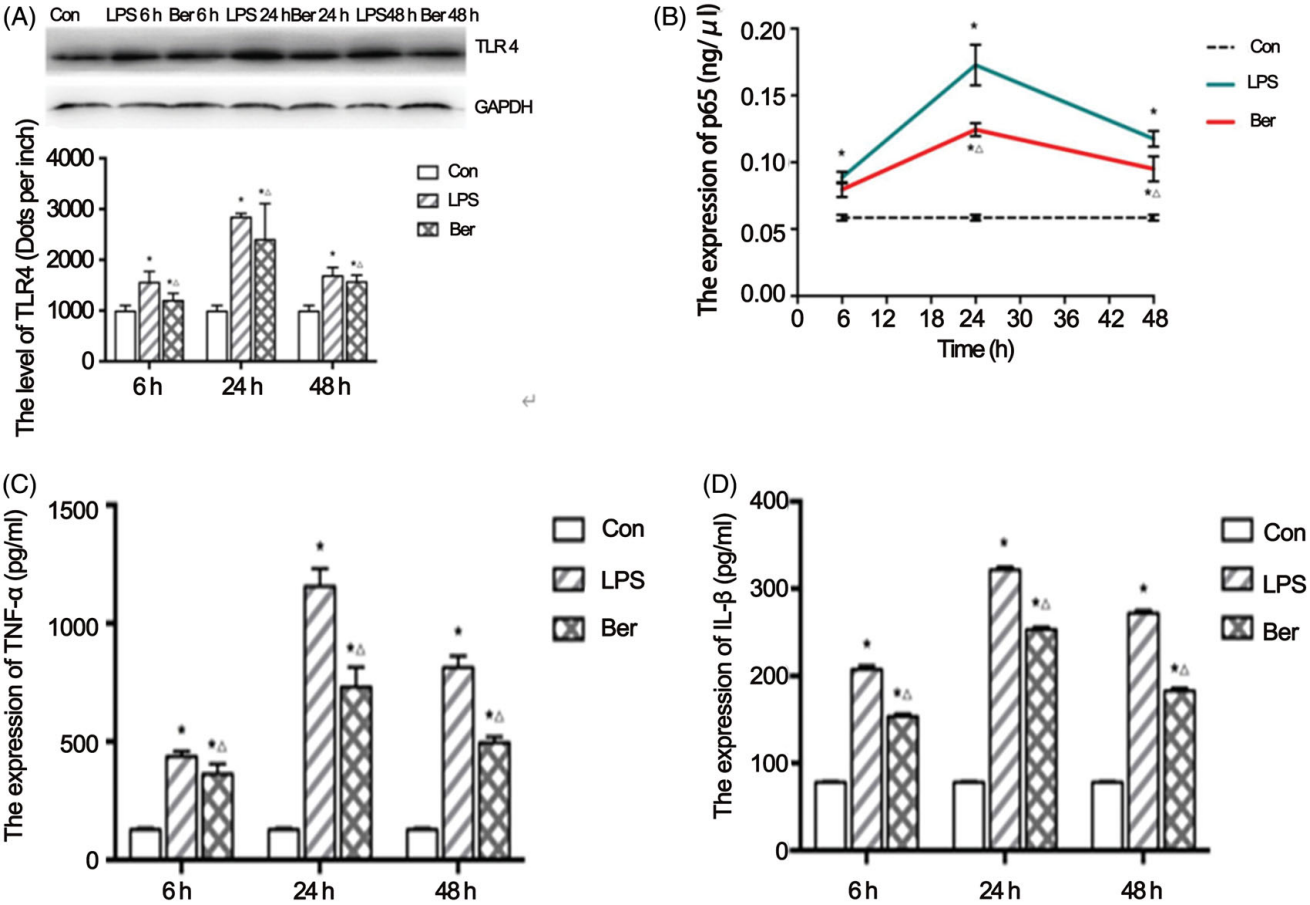
Effects of berberine on key effectors of the TLR4/NF-κB signalling pathway. (A) TLR4 protein expression in the myocardial tissue and quantitation. (B) Nuclear p65 protein expression in the rat sepsis cardiomyopathy model measured by ELISA. Effects of berberine on TNFα (C) and IL 1β (D) levels in the myocardial tissue. *p < 0.001 indicates significance in comparison with the Con group. △*p* < 0.001 indicates significance in comparison with the LPS group.

WB was performed to evaluate the expression of a key protein, TLR4, in the myocardial tissue of sepsis rats. The results showed that TLR4 protein expression was upregulated significantly in the LPS group (1550.53 ± 219.49, 2834.60 ± 81.02, 1682.93 ± 170.38 dots per inch) at 6, 24, and 48 h, compared with that in the Con group (985.30 ± 114.87 dots per inch at 6 h) (all *p* < 0.05). Berberine pre-treatment significantly decreased LPS-induced TLR4 expression at each individual time point (1196.43 ± 145.24, 2393.70 ± 713.20, 1561.30 ± 137.56 dots per inch at 6, 24, and 48 h, correspondingly) (all *p* < 0.05) (Figure 3(A)). These findings suggested that Ber reduced LPS-induced TLR4 upregulation in the myocardial tissue.

### Effects of berberine on the NF-κB signalling pathway in LPS-induced cardiomyocytes

To investigate the effects of berberine on the NF-κB signalling pathway in LPS-induced cardiomyocytes, the nuclear p65 protein expression was measured by ELISA. The expression levels of p65 in the LPS group were significantly higher (0.089 ± 0.004 ng/μL, 0.173 ± 0.015 ng/μL, 0.118 ± 0.006 ng/μL at 6, 24, and 48 h, correspondingly) than that in the Con group (0.59 ± 0.02 ng/μL at 6 h) (all *p* < 0.05), and significantly decreased in the Ber group (0.079 ± 0.006 ng/μL, 0.124 ± 0.005 ng/μL, 0.095 ± 0.009 ng/μL at 6, 24, and 48 h, correspondingly) compared with that in the LPS group (all *p* < 0.05) (Figure 3(B)). Thus, berberine likely inhibited the activation of NF-κB in myocardial tissues of septic rats.

### TNF-α and IL-1β levels are significantly increased in the LPS group but reduced by Ber treatment

ELISA was performed to investigate the effects of berberine on the expression of TNF-α and IL-1β levels in the myocardial tissue. Significantly increased levels of TNF-α (437.20 ± 22.00 pg/mL, 1155.49 ± 75.09 pg/mL, 730.35 ± 84.47 pg/mL) and IL-1β (207.50 ± 4.19 pg/mL, 321.59 ± 3.14 pg/mL, 271.79 ± 3.53 pg/mL) were observed in both the LPS and Ber groups (TNF-α: 363.12 ± 42.18 pg/mL, 812.92 ± 48.79 pg/mL, 495.83 ± 25.21 pg/mL; IL-1β: 153.54 ± 3.05 pg/mL, 253.25 ± 2.85 pg/mL, 182.72 ± 3.32 pg/mL) at 6, 24, and 48 h, correspondingly, compared with that in the Con group (TNF-α: 127.94 ± 6.06 pg/mL, IL-1β: 78.22 ± 0.85 pg/mL) at 6 h (all *p* < 0.05). However, the Ber group showed significantly decreased levels of TNF-α and IL-1β at each time point compared with the LPS group (all *p* < 0.05) (Figure 3(C, D)). These results suggested that berberine alleviated TNF-α and IL-1β upregulation in the myocardial tissue of septic rats.

### Correlation of myocardial injury with NF-κB signalling

**Figure 4.**
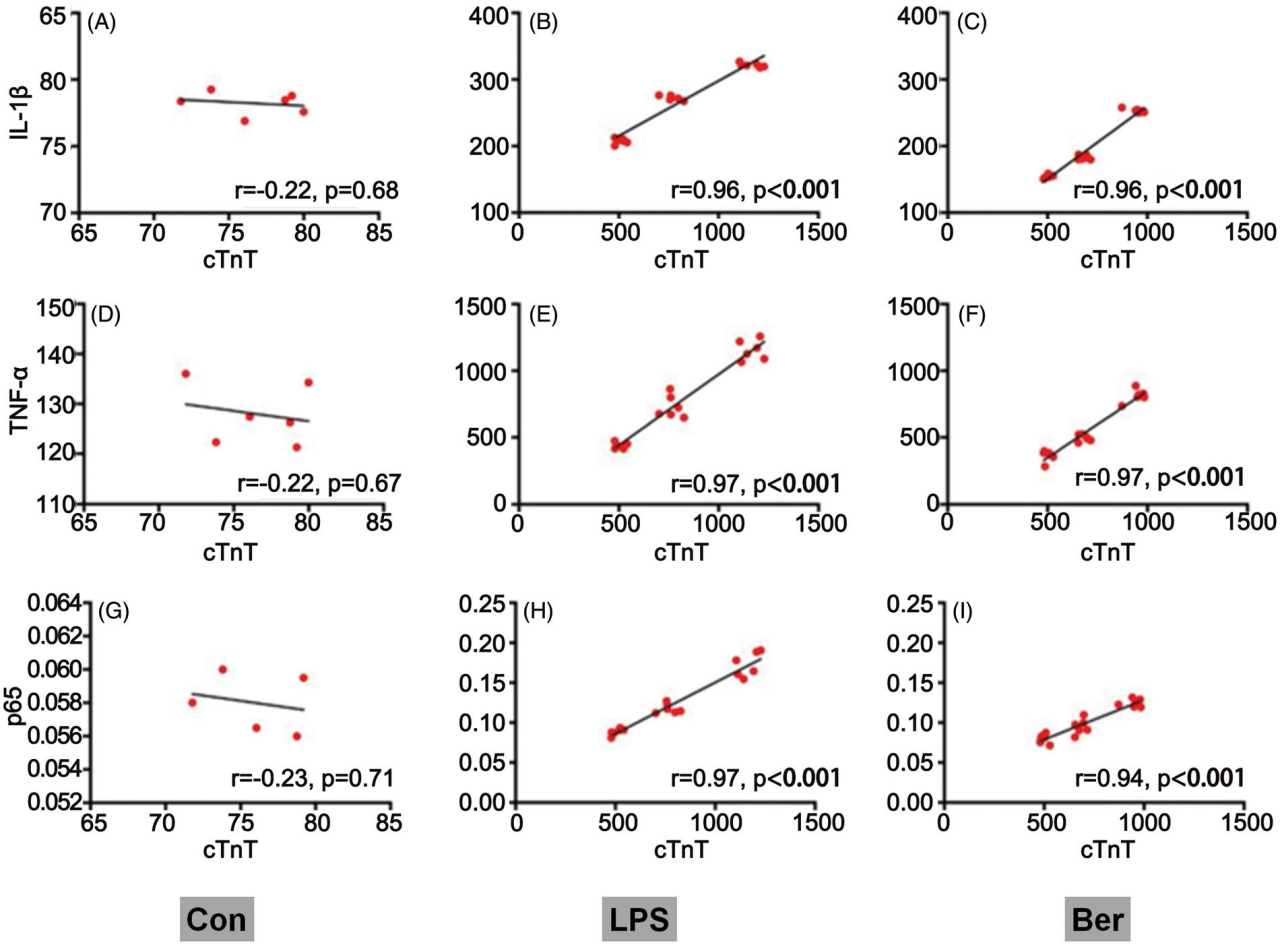
Correlation between cTnT and NF-κB signalling. Associations of cTnT amounts with IL-1β, TNFα and p65 levels in the Con (A, B, C), LPS (D, E, F) and Ber (G, H, I) groups, assessed by Pearson correlation analysis r correlation coefficient. *p* < 0.05 indicates a valid correlation.

Associations of plasma cTnT amounts with the expression levels of key effectors (IL-1β, p65, and TNF-α) in the NF-κB signalling pathway were analysed. The results showed that cTnT was not correlated with p65, TNF-α and IL-1β in the control group (Figure 4(A)). After LPS induction, cTnT amounts were positively associated with p65, TNF-α and IL-1β consistently resulted in a positive correlation (*p* < 0.001) (Figure 4(B)). In the Ber group, cTnT amounts also showed positive correlations with the key signalling molecules p65, TNF-α and IL-1β (*p* < 0.001, Figure 4(C)). Taken together, NF-κB signalling is involved in sepsis-induced myocardial injury.

## Discussion

Septic cardiomyopathy (SCM) is often diagnosed when some acute perturbation in cardiac function exists in the setting of sepsis, it is the most common type of sepsis-related organ dysfunction. Some studies suggest that mortality is two to three times greater when SCM is present (Ehrman et al. 2018). At present, no formalized or consensus precise definition of SCM exists, representing a critical knowledge gap. Martin et al. (2019) suggest defining septic cardiomyopathy broadly as a sepsis-associated acute syndrome of cardiac dysfunction unrelated to ischaemia with one or more of the main characteristics: (1) left ventricular dilatation with normal- or low-filling pressure, (2) reduced ventricular contractility, and (3) right ventricular dysfunction or left ventricular (systolic or diastolic) dysfunction with a reduced response to volume infusion. The main challenges in this definition are the evaluation of the cardiovascular context, and the lack of longitudinal echocardiography data starting from premorbid heart function with serial echocardiographic evaluations performed during the course of the critical illness and eventually following recovery (Sanfilippo et al. 2019). Several mechanisms have been proposed to explain the pathophysiology of septic cardiomyopathy, including excessive formation of nitric oxide (NO), reactive oxygen species (ROS) or nitrogen radicals, and transcriptional and metabolic changes (Kakihana et al. 2016). But the specific pathogenesis of SCM has not been fully elucidated, and new discoveries in this field may help find novel therapeutic targets and allow the development of effective treatments (Potz et al. 2016; Gyongyosi et al. 2017).

Berberine (Ber) is a natural isoquinoline alkaloids extracted from rhizomes of *Coptis chinensis* Franch (Ranunculaceae), *Phellodendron* (Rutaceae), and other plants in the genus *Coptis*. It’s a nitrogen-containing low-molecular-weight compound, appearing as yellow needle-like crystals with bitter taste and high biological activity. Ber is mainly used as an antibacterial drug to treat gastrointestinal infections such as diarrhoea, dysentery, and malaria. Modern pharmacological studies have found that Ber also has antitumor, lowering blood sugar, protection of cardiovascular and cerebrovascular, anti-inflammatory, anti-Alzheimer’s disease, anti-arrhythmic, and antidepressant effects (Sun and Wu 2020). Recent studies have found that berberine increases the survival rate of sepsis mice by affecting the extracellular release of high mobility group box 1 (HMGB1) (Lee et al. 2013). It was also found to inhibit LPS-induced cardiac dysfunction (Denk et al. 2012). However, the role of berberine in protecting SCM remains unknown. In this study, berberine was demonstrated to exert a protective effect in septic cardiomyopathy rats possibly by inhibiting the TLR4/NF-κB signalling pathway.

LPS is commonly used to establish sepsis models characterized by ventricular dilatation, decreased left ventricular ejection fraction and cardiac dysfunction found in SCM (Parker et al. 1984). In this study, the SCM rat model was established by intraperitoneal injection of LPS and evaluated by assessing cardiac function, myocardial injury, and morphological changes in the myocardial tissue (Chen et al. 2017). The results showed that myocardial systolic function indicators (LVSP and + dp/dt max) and myocardial diastolic indicator (–dp/dt max) were decreased, LVEDP and the plasma myocardial injury marker cTnT were increased. In addition, cardiomyocyte injury and inflammatory cell infiltration in the myocardial tissue became more severe as determined by H&E staining. Therefore, SCM was successfully induced in this study.

In these experiments, berberine was found to alleviate myocardial injury in SCM, as cTnT levels were significantly reduced. Furthermore, cTnT was positively correlated with the relative expression levels of p65 level in the nucleus of myocardial cells. Previous studies have shown that in the early stage of sepsis, TLR4 activation is triggered under the stimulation of inflammatory mediators and endotoxins to induce the downstream NF-κB signalling pathway (Parker et al. 1984; Kakihana et al. 2016; Lv and Wang 2016). It was therefore speculated that berberine could reduce myocardial damage by inhibiting the TLR4/NF-κB signalling pathway.

TLR4 activation is frequently accompanied by excessive release of septic myocardial injury-related pro-inflammatory cytokines, including IL-1, IL-6 and TNF-α (Kakihana et al. 2016; Lv and Wang 2016). In this study, berberine not only reduced TLR4 upregulation, but also alleviated the increases of TNF-α and IL-1β amounts in the myocardial tissue in the SCM rat model. Inflammatory factors such as IL-6, IL-1β and TNF-α play key roles in SCM induced by sepsis (Wu et al. 2015). Our results are consistent with previous findings that berberine could suppress NF-κB activation induced by various inflammatory agents with a mild potency (Pandey et al. 2008).

Modern pharmacological studies have found that berberine (50 mg/kg) is distributed in many tissues after oral administration, such as heart, spleen, liver, kidney, brain, intestine, muscle and fat. Except for the intestinal concentration of 4000 ng/g, other tissues or organ concentration is 200 ng/g, the concentration of berberine in plasma has an obvious non-linear relationship with the oral dose (Chen and Lu 2020). The underlying molecular mechanism of berberine pharmacological activation is not yet fully understood. Research has shown that mitochondria play an important role in organ damage during Sepsis, the mitochondria-related mechanisms in septic cardiomyopathy have been discussed in terms of restoring mitochondrial function (Pan et al. 2018).

## Conclusions

Our study showed that berberine reduces myocardial injury, and enhances myocardial contraction and diastolic function to alleviate SCM in sepsis rats, by inhibiting sepsis-induced TLR4/NF-κB signal pathway activation and decreasing the expression levels of TNF-α, IL-1β and other inflammatory factors. Berberine therefore constiutes a potential drug for SCM treatment. Further studies assessing whether berberine alleviates SCM by regulating other mechanisms, e.g., myocardial mitochondrial dynamics, are required to further elucidate the complicate pathogenesis of SCM. Berberine should also be further studied for its pharmacokinetic and pharmacodynamic parameters.

## Author contributions

Chen Huiqi performed data acquisition. Liu Qian contributed to data acquisition, analysis and interpretation. Liu Xiangqi was involved in manuscript drafting. Jin jinlan performed study conception and design, revised the manuscript, and gave final approval of the version to be published.
